# An Improved Method for Detecting Crane Wheel–Rail Faults Based on YOLOv8 and the Swin Transformer †

**DOI:** 10.3390/s24134086

**Published:** 2024-06-24

**Authors:** Yunlong Li, Xiuli Tang, Wusheng Liu, Yuefeng Huang, Zhinong Li

**Affiliations:** 1 Beijing Materials Handling Research Institute Co. Ltd., Beijing 100007, China; 2 Department of Industrial Engineering, Tsinghua University, Beijing 100084, China; 3 Key Laboratory of Nondestructive Testing of the Ministry of Education, Nanchang Hangkong University, Nanchang 330063, China

**Keywords:** fault detection, Swin Transformer, YOLOv8, special equipment, feature pyramid network

## Abstract

In the realm of special equipment, significant advancements have been achieved in fault detection. Nonetheless, faults originating in the equipment manifest with diverse morphological characteristics and varying scales. Certain faults necessitate the extrapolation from global information owing to their occurrence in localized areas. Simultaneously, the intricacies of the inspection area’s background easily interfere with the intelligent detection processes. Hence, a refined YOLOv8 algorithm leveraging the Swin Transformer is proposed, tailored for detecting faults in special equipment. The Swin Transformer serves as the foundational network of the YOLOv8 framework, amplifying its capability to concentrate on comprehensive features during the feature extraction, crucial for fault analysis. A multi-head self-attention mechanism regulated by a sliding window is utilized to expand the observation window’s scope. Moreover, an asymptotic feature pyramid network is introduced to augment spatial feature extraction for smaller targets. Within this network architecture, adjacent low-level features are merged, while high-level features are gradually integrated into the fusion process. This prevents loss or degradation of feature information during transmission and interaction, enabling accurate localization of smaller targets. Drawing from wheel–rail faults of lifting equipment as an illustration, the proposed method is employed to diagnose an expanded fault dataset generated through transfer learning. Experimental findings substantiate that the proposed method in adeptly addressing numerous challenges encountered in the intelligent fault detection of special equipment. Moreover, it outperforms mainstream target detection models, achieving real-time detection capabilities.

## 1. Introduction

Special equipment holds paramount significance in contemporary society, manifesting not only within industrial production, construction and transportation but also across diverse societal levels. These devices, comprising construction cranes [1], ropeways [2], forklifts [3], pressure vessels [4] and so on, provide essential support and protection for human production activities. Typically, special equipment operates in complex and harsh environments characterized by extremes in temperature, high humidity, corrosive gases and other challenging conditions. Prolonged exposure to these conditions results in the equipment being subject to wear, corrosion, and subsequent failure. Consequently, diagnosing faults in special equipment holds immense significance.

Machine vision offers notable advantages in diagnosing faults of special equipment [5,6]. With the help of deep learning and image processing technology, detecting and diagnosing equipment faults quickly and accurately is achieved by utilizing images and video. Target detection algorithms rooted in deep learning are pivotal within the realm of computer vision, categorized primarily into two major frameworks: two-stage frameworks [7,8,9] and single-stage frameworks [10,11,12]. The core concept of the two-stage framework involves dividing the target detection task into two key stages: initial coarse classification and refined position adjustment. This framework exhibits unique advantages in addressing the issue of non-uniform fault categories in special equipment. On the other hand, the single-stage framework employs an end-to-end approach to directly predict the category and location of the target through a unified neural network model. The single-stage framework usually offers faster processing with a simpler structure. YOLO [13] is a representative single-stage target detection algorithm. It might encounter issues such as missed detection or imprecise localization when handling small objects or densely populated target areas within a scene. Therefore, recent advancements in the YOLO algorithm, namely YOLOv3 [14], YOLOv4 [15] and subsequent versions [16,17,18,19], aim to enhance detection accuracy and robustness by implementing novel techniques and optimization strategies, thereby striving for improved performance across diverse scenarios. The recently introduced YOLOv8 [20] adopts a streamlined model structure, augmented convolutional kernels, accelerated convolutional operations and a more concise architecture, resulting in swifter inference compared to its predecessors in the YOLO series. As a result, YOLOv8 has been applied in various fields [21,22,23]. Luo et al. [24] revamped a novel lightweight ShuffleNetV2 network, integrating it as the backbone of the YOLOv8 target detection network. Additionally, they introduced a simple and parameter-free attention mechanism. The proposed model boasts fewer redundant parameters and demonstrates improved precision in recognizing foreign object features. Ye and Chen [25] incorporated the Ghost module into YOLOv8, enhancing the backbone’s feature extraction and reducing the model’s computational load. Furthermore, they devised a bidirectional omni-dimensional dynamic neck for YOLOv8 to weigh and merge feature information across layers in intricate logistics scenarios.

Nevertheless, YOLOv8’s limited robustness to small targets, low-quality images or intricate scenes impedes its direct application in fault detection within special equipment. The primary factor is that YOLOv8 incorporates multiple CNN layers without global attention mechanisms. Over recent years, the emergence of Transformers [26] has addressed the shortcomings of CNN and RCNN in global feature extraction, sparking a revolution in natural language processing. Transformers have found extensive applications, notably in the widely acclaimed ChatGPT. Simultaneously, the adoption of enhanced Transformer models in computer vision is steadily gaining traction [27,28]. To enhance the Transformer’s image processing capabilities, Microsoft Research Asia has introduced a Transformer model with a hierarchical structure, known as Swin Transformer (ST) [29,30]. ST partitions the input image into numerous subgraphs, employing the Transformer model on each. Unlike conventional global attention, ST confines attention to the local region and captures fault feature information utilizing a moving window. By employing this attention mechanism, the model becomes more adept at capturing local spatial information in the fault feature image, enhancing its capacity to model spatial information.

Additionally, YOLOv8 incorporates a feature pyramid network (FPN) structure built by the path aggregation network (PANet) [31]. PANet aggregates feature maps across various levels using horizontal and vertical paths, thereby augmenting the model’s capability to detect objects at diverse scales. Through improved network information utilization, connections between feature maps at different levels are established, thereby enhancing the expression of semantic-level features. However, the propagation and interaction process of PANet might lead to the degradation or loss of semantic information from higher-level features. An asymptotic feature pyramid network (AFPN) is proposed to preserve all features consistently during feature extraction, iteratively combining low-level and high-level features to create enriched low-level features. During the bottom-up feature extraction within the backbone network, two low-level features of differing resolutions are merged in the initial stage to prevent semantic disparities between disparate levels. High-level features gradually merge into the process in the later stages, ultimately contributing to the formation of the top-level features of the backbone. Low-level features integrate the semantic information of high-level features, while high-level features incorporate the detailed information from low-level features. The direct interaction between low-level and high-level features prevents information degradation or loss during multi-level transmission.

Consequently, we propose an enhanced YOLOv8 network for diagnosing faults in special equipment, leveraging the strengths of ST. The model enhances the fusion of fault features in special equipment across various scales, prioritizing the fault region. The model allows for improved utilization of global information in fault inference, resulting in superior detection performance, especially for small target faults. As a case study, we apply the proposed method for diagnosing wheel-rail faults in lifting equipment. The main contributions of this paper are as follows.

(1) Building upon the single-stage framework of YOLOv8, the ST is integrated with the network structure of YOLOv8 to augment the model’s capability for extracting global information.
(2) To avoid the loss or degradation of feature information during transmission and interaction, the AFPN-based YOLOv8 model is proposed to fuse features from non-adjacent layers directly.
(3) Recognizing the similarity between the fault features of wheel-rails in lifting equipment and railway systems, a transfer learning strategy [32] is employed to augment the lifting equipment wheel-rail fault dataset. The model undergoes pre-training using the railway wheel-rail fault dataset, and subsequently, the trained model is transferred to the lifting equipment wheel-rail fault dataset. This approach expedites the convergence speed of the proposed algorithm, thereby enhancing the accuracy and robustness of the lifting equipment wheel-rail fault diagnosis, especially in cases with limited samples.

The subsequent sections of the paper are organized as follows. Section 2 provides an in-depth explanation of the proposed methodology designed for effectively detecting and identifying faults in special equipment. Section 3 is dedicated to presenting the experimental results and their analysis. Lastly, Section 4 summarizes and presents the conclusions drawn from this study.

## 2. Proposed Methodology

YOLOv8 utilizes a single neural network model to swiftly and accurately detect and localize multiple targets within an image. Leveraging the simple and lightweight CSPDarkNet-53 network as the basis for the backbone network, YOLOv8 enables the achievement of rapid real-time target detection, particularly suited for scenarios necessitating efficient processing of numerous images. The backbone network comprises five ConvModule modules and four CSPLayer_2Conv modules, each housing multiple CNN layers that excel at capturing local information [33]. However, unlike CNN, the Transformer is not constrained by local interactions, due to its self-attention mechanism allowing parallel computation. The Transformer’s prowess in fault diagnosis stems from exceptional sequence modeling and automatic focus on fault characteristics. While exhibiting performance enhancement in diagnosing faults compared to traditional methods, the Transformer model retains many parameters and lacks spatial information modeling capabilities. Fortunately, ST enhances efficiency by confining self-attention computations to a localized window, ensuring versatile modeling across different scales while leaving the other layers unchanged.

### 2.1. Swin Transformer

**Figure 1:**
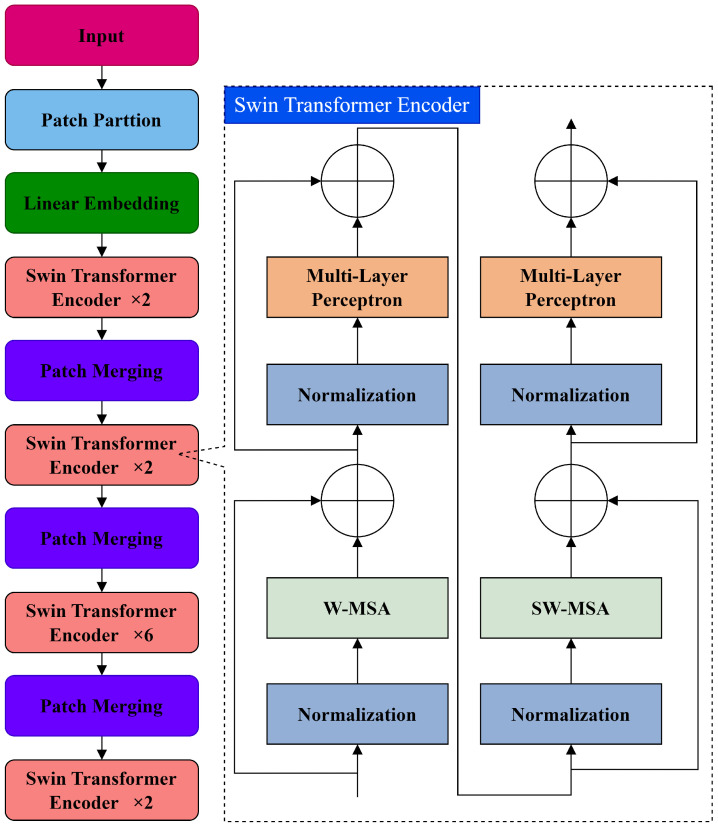
Swin Transformer network structure.

ST serves as the foundational visual network for tasks, employing a self-attention mechanism. ST’s structure is depicted in Figure 1. The structure primarily comprises a patch partition layer, a linear embedding layer, patch merging layers, and ST encoder layers.

Initially, ST divides the input image into non-overlapping patches using the patch partition layer, where each patch’s features are defined as a sequence of original pixel values. The linear embedding layer projects a feature mapping $X\in R^{H\times W\times C}$ into arbitrary dimensions $C^{\prime }$, resulting in the creation of query vector *Q*, key vector *K*, and value vector *V* ($Q,K,V\in R^{H\times W\times C^{\prime }}$). The multi-head self-attention (MSA) mechanism, central to the Transformer encoder, allocates varying weights based on the significance of specific image regions, allowing the network to prioritize key information for aligning the extracted features with detected targets. Following scaling and Softmax normalization by a certain factor, the semantic weights derive from the multiplication of *V* by the similarity value obtained from the dot product of *K* and *Q*. Subsequently, these semantic weights are added to the image, contributing to the generation of self-attentive features achieved by weighting and summing all semantic weights. The formula for the self-attention mechanism is

(1) Z=AV

(2) $$A=\mathrm{Softmax}(\frac{QK^{T}}{\sqrt{d}})$$

where *Z* is the self-focused feature, *A* is the similarity value of *K* and *Q*, and *d* is the scaling factor.

Global computation introduces secondary complexities linked to patch numbers and is unsuitable for intensive prediction tasks. To address this, ST modifies the self-attention computation to focus on local windows within the ST encoder, incorporating normalization layer, window-based MSA (W-MSA), shift window-based MSA (SW-MSA), and multi-layer perceptron. The W-MSA module divides the input image into non-overlapping small windows and computes self-attention separately within these windows. Therefore, the computational complexity Ω for localized windows is

(3) $$\Omega_{\mathrm{MSA}}=4HWC^{2}+2{\left(HW\right)}^{2}C$$

(4) $$\Omega_{W\text{-}\mathrm{MSA}}=4HWC^{2}+2HWM^{2}C$$

where *M* denotes small windows contained in windows.

While W-MSA reduces the complexity of self-attention computation, it faces difficulty extracting high-level semantic information due to limited interaction between small windows. The SW-MSA module resolves this by connecting upper layers of adjacent non-overlapping small windows, expanding the receptive field to capture richer semantic information from the image. Consequently, ST necessitates an even number of consecutive encoders alternating between the W-MSA and SW-MSA modules. Geometric relations in self-attentive computation are encoded through the introduction of additional parameter bias terms, i.e.,

(5) $$Z=\mathrm{Attention}\left(Q,K,V\right)=\mathrm{Softmax}\left(\frac{QK^{T}}{\sqrt{d}}+B\right)V$$

where $B\in R^{H\times W}$ represents each head’s relative position bias term. The relative position bias plays a crucial role in encoding spatial configurations among visual elements, particularly in dense recognition tasks like object detection.

Aiming to create a hierarchical representation with the network’s increasing depth, merging layers is necessary to reduce the feature count. Subsequently, the ST encoder is applied to transform these features.

### 2.2. Asymptotic Feature Pyramid Network

**Figure 2:**
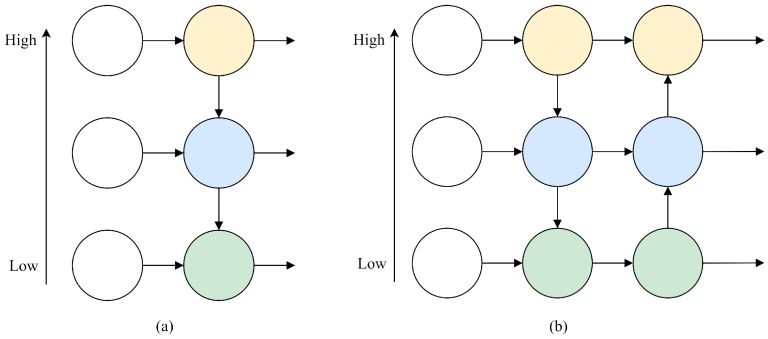
Structure of FPN and PANet: (**a**) FPN. (**b**) PANet.

Moreover, YOLOv8 incorporates the path aggregation network (PANet) feature pyramid structure. As shown in Figure 2, high-level features situated at the apex of FPN must traverse multiple intermediate scales, interacting with features at these scales before amalgamating with the low-level features at the base. Semantic information from high-level features could be lost or diminished throughout this propagation and interaction process. Conversely, PANet employs upsampling to pass and fuse feature information from higher levels, facilitating the generation of feature maps for prediction and the transfer of semantic information from higher to lower dimensions. Feature information is allocated across various layers based on the network’s size, assigning smaller features to lower layers and larger features to higher layers. Consequently, YOLOv8 achieves more accurate detection of sizes, shapes, and classes of targets. However, despite YOLOv8’s simplicity and effectiveness, the bottom-up trajectory of PANet presents an inverse challenge, wherein detailed information from low-level features might degrade or be lost during propagation and interaction.

**Figure 3:**
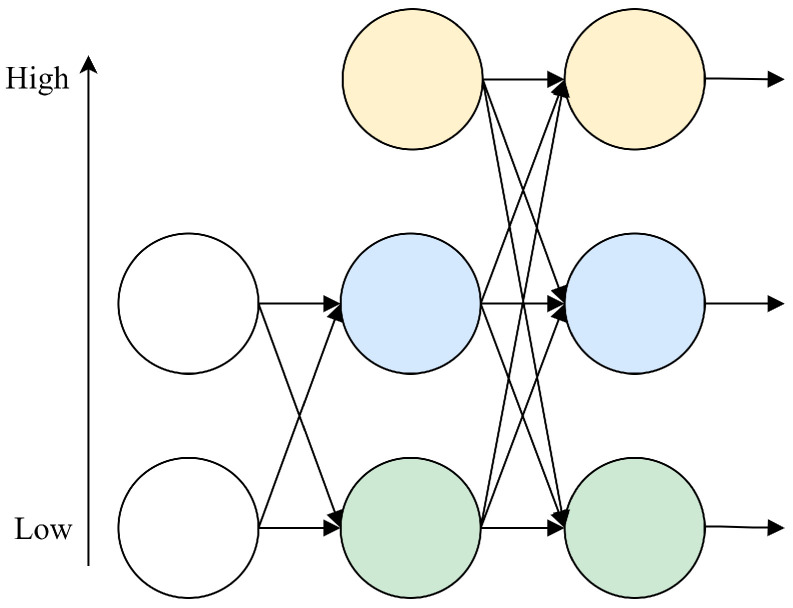
AFPN structure.

We observe that the high-resolution network [34] consistently maintains low-level features throughout the feature extraction process, iteratively merging them with high-level features to enrich the low-level features’ depth. Motivated by the architecture of the high-resolution network, we introduce the AFPN to overcome this restriction, as depicted in Figure 3. During the bottom-up feature extraction within the backbone, the fusion process commences by amalgamating two low-level features with distinct resolutions at the initial stage. Subsequently, as progression occurs to later stages, the integration of high-level features incrementally contributes to the fusion process, culminating in the fusion of the backbone’s top-level features. This fusion methodology mitigates semantic disparities among disparate levels. Within this process, low-level features integrate semantic information from high-level features, while high-level features assimilate detailed information from low-level features. Direct interaction between these features mitigates the risk of information loss or degradation during multilevel transmission.

Element-by-element summation proves ineffective within the entire feature fusion process due to potential contradictions arising from different objects across levels at a given location. Addressing this issue, we employ adaptively spatial feature fusion (ASFF) [35] to allocate distinct spatial weights to features across various levels during the multilevel feature fusion process, aiming to amplify the significance of pivotal levels while minimizing the influence of conflicting information originating from diverse objects. Consider $X_{ij}^{n\to m}$ as the feature vector transitioning from level *n* to level *m* at the position (i,j), while the resulting resultant vector, denoted as $Y_{ij}^{m}$, is derived through multilevel ASFF. The resulting feature vector is formulated as a linear combination of the feature vectors $X_{ij}^{1\to m}$, $X_{ij}^{2\to m}$ and $X_{ij}^{3\to m}$, defined as follows [36].

(6) $$Y_{ij}^{m}=\alpha_{ij}^{m}\cdot X_{ij}^{1\to m}+\beta_{ij}^{m}\cdot X_{ij}^{2\to l}+\gamma_{ij}^{m}\cdot X_{ij}^{3\to m}$$

where $Y_{ij}^{m}$ represents the (i,j)th vector within the output feature map $Y^{m}$ across channels; and $\alpha_{ij}^{m}$, $\beta_{ij}^{m}$ and $\gamma_{ij}^{m}$ denote spatial importance weights for the feature maps from various levels to level *m*, dynamically learned by the network. It is important to note that $\alpha_{ij}^{m}$, $\beta_{ij}^{m}$ and $\gamma_{ij}^{m}$ can function as simple scalar variables, shared across all channels. Drawing inspiration from adaptively connected neural networks [37], we define $\alpha_{ij}^{m}=\frac{e^{\lambda_{\alpha_{ij}}^{m}}}{e^{\lambda_{\alpha_{ij}}^{m}}+e^{\lambda_{\beta_{ij}}^{m}}+e^{\lambda_{\gamma_{ij}}^{m}}}$, resulting in

(7) $$\alpha_{ij}^{m}+\beta_{ij}^{m}+\gamma_{ij}^{m}=1$$

where $\alpha_{ij}^{m}$, $\beta_{ij}^{m}$ and $\gamma_{ij}^{m}$ are defined by Softmax functions with control parameters defined as $\lambda_{\alpha_{ij}}^{m}$, $\lambda_{\beta_{ij}}^{m}$ and $\lambda_{\gamma_{ij}}^{m}$.

According to the chain rule, the gradient of Equation (6) is calculated as follows.

(8) $$\frac{\partial L}{\partial X_{ij}^{1}}=\frac{\partial Y_{ij}^{1}}{\partial X_{ij}^{1}}\cdot \frac{\partial L}{\partial Y_{ij}^{1}}+\frac{\partial X_{ij}^{1\to 2}}{\partial X_{ij}^{1}}\cdot \frac{\partial Y_{ij}^{2}}{\partial X_{ij}^{1\to 2}}\cdot \frac{\partial L}{\partial Y_{ij}^{2}}+\frac{\partial X_{ij}^{1\to 3}}{\partial X_{ij}^{1}}\cdot \frac{\partial Y_{ij}^{3}}{\partial X_{ij}^{1\to 3}}\cdot \frac{\partial L}{\partial Y_{ij}^{3}}$$

Notably, feature extraction commonly employs interpolation for upsampling and pooling for downsampling. To simplify, we assume that $\frac{\partial X_{ij}^{1\to m}}{\partial X_{ij}^{1}}\approx 1$. Consequently, Equation (8) can then be expressed as

(9) $$\frac{\partial L}{\partial X_{ij}^{1}}=\frac{\partial Y_{ij}^{1}}{\partial X_{ij}^{1}}\cdot \frac{\partial L}{\partial Y_{ij}^{1}}+\frac{\partial Y_{ij}^{2}}{\partial X_{ij}^{1\to 2}}\cdot \frac{\partial L}{\partial Y_{ij}^{2}}+\frac{\partial Y_{ij}^{3}}{\partial X_{ij}^{1\to 3}}\cdot \frac{\partial L}{\partial Y_{ij}^{3}}$$

For the two commonly used fusion operations (element-by-element summation and cascade), the equation can be further reduced to the following equation with $\frac{\partial Y_{ij}^{1}}{\partial X_{ij}^{1}}=1$ and $\frac{\partial Y_{ij}^{m}}{\partial X_{ij}^{1\to m}}=1$.

(10) $$\frac{\partial L}{\partial X_{ij}^{1}}=\frac{\partial L}{\partial Y_{ij}^{1}}+\frac{\partial L}{\partial Y_{ij}^{2}}+\frac{\partial L}{\partial Y_{ij}^{3}}$$

Following the scale matching mechanism, the position (i,j) at level 1 is identified as the object’s centre, where $\frac{\partial L}{\partial Y_{ij}^{1}}$ represents the gradient from the positive sample. As the corresponding positions are treated as background in other levels, $\frac{\partial L}{\partial Y_{ij}^{2}}$ and $\frac{\partial L}{\partial Y_{ij}^{3}}$ represent the gradient from the negative sample. Such inconsistency impacts the gradient of $\frac{\partial L}{\partial X_{ij}^{1}}$, subsequently diminishing the training efficiency of the original feature map.

A common approach to address this issue involves designating the corresponding positions of the other levels as ignore regions, effectively setting $\frac{\partial L}{\partial Y_{ij}^{2}}=\frac{\partial L}{\partial Y_{ij}^{3}}=0$.

For ASFF, the gradient can be computed directly from Equations (6) and (9) as follows.

(11) $$\frac{\partial L}{\partial X_{ij}^{1}}=\alpha_{ij}^{1}\cdot \frac{\partial L}{\partial Y_{ij}^{1}}+\alpha_{ij}^{2}\cdot \frac{\partial L}{\partial Y_{ij}^{2}}+\alpha_{ij}^{3}\cdot \frac{\partial L}{\partial Y_{ij}^{3}}$$

where $\alpha_{ij}^{1}$, $\alpha_{ij}^{2}$, and $\alpha_{ij}^{3}$ are within the range of [0, 1]. The convergence of $\alpha_{ij}^{2}$ and $\alpha_{ij}^{3}$ towards 0 facilitates the reconciliation of gradient inconsistency by utilizing these three coefficients. Given that fusion parameters are trainable by standard backpropagation algorithms, a meticulously tuned training process can yield these effective coefficients.

### 2.3. Structure of the Proposed Methodology

**Figure 4:**
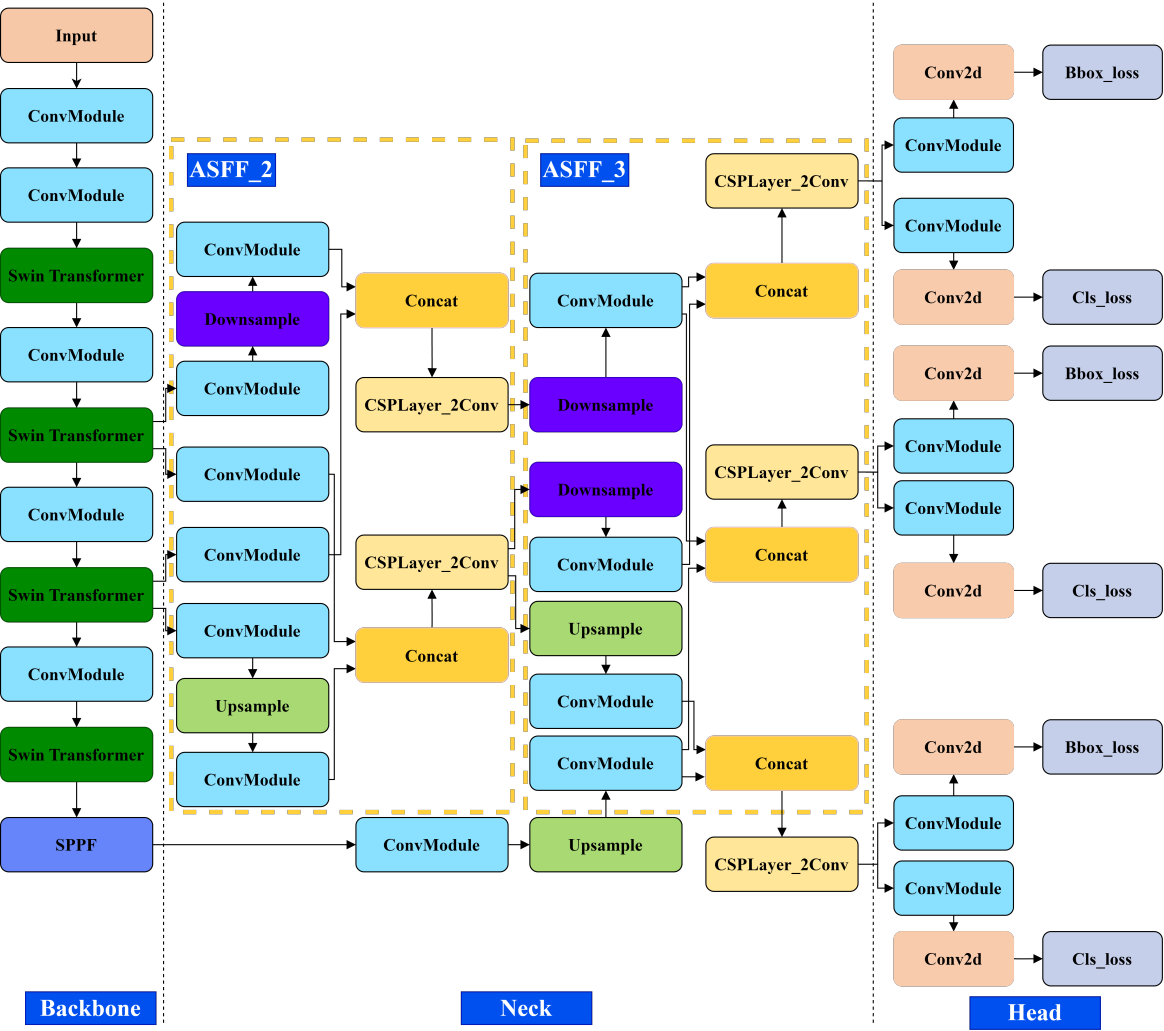
Structure of the proposed methodology.

Consequently, we propose an enhanced YOLOv8 network for detecting faults in special equipment, leveraging the benefits of the ST. Based on the AFPN model, the network achieves direct feature fusion across non-adjacent layers. Figure 4 illustrates the architecture of the proposed method.

### 2.4. Fault Diagnosis Framework Based on the Proposed Method

**Figure 5:**
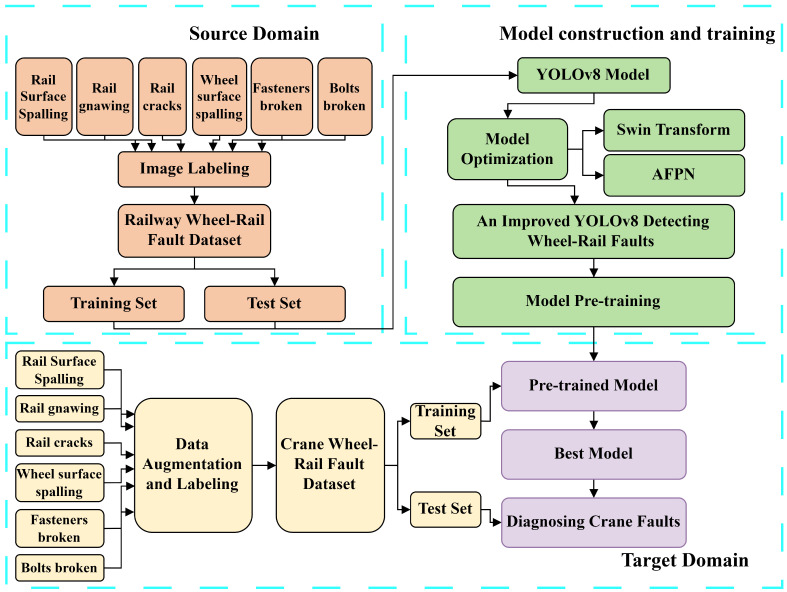
Flow diagram of the proposed method detecting faults in special equipments.

According to the idea of transfer learning, the proposed diagnostic method for crane wheel–rail faults uses YOLOv8 as the base diagnostic model for fault feature extraction from the input image, and its flowchart is shown in Figure 5. The proposed method makes full use of the advantages of YOLOv8 and Swin Transformer in the field of image processing in order to realize the accurate identification and classification of faults. The diagnostic process of the proposed method includes the following steps.

(1) Fault images of publicly available figures of railroad wheel–rail are used as source domain data, which are preprocessed and constructed into a dataset.
(2) The source domain dataset is utilized to train the fault diagnosis model based on the improved YOLOv8 model in order to obtain the trained source domain fault diagnosis model.
(3) Fault images of the crane wheel–rail are used as target domain data, and the dataset is constructed after preprocessing them by flipping, rotating and radiating.
(4) The pretrained model of the source domain is retrained using the target domain dataset with fine-tuned updates to the network parameters and weights. The optimized target domain diagnostic model is highly adaptive to the target domain data.
(5) After the training of the target domain diagnostic model is completed, the model is tested using the test dataset. The diagnostic performance of the model is evaluated by the final diagnostic accuracy.

## 3. Experiments and Results Analysis

In this section, we conduct comprehensive training of the enhanced YOLOv8 model using the dataset comprising instances of lifting equipment faults.

### 3.1. Experimental Datasets

**Figure 6:**
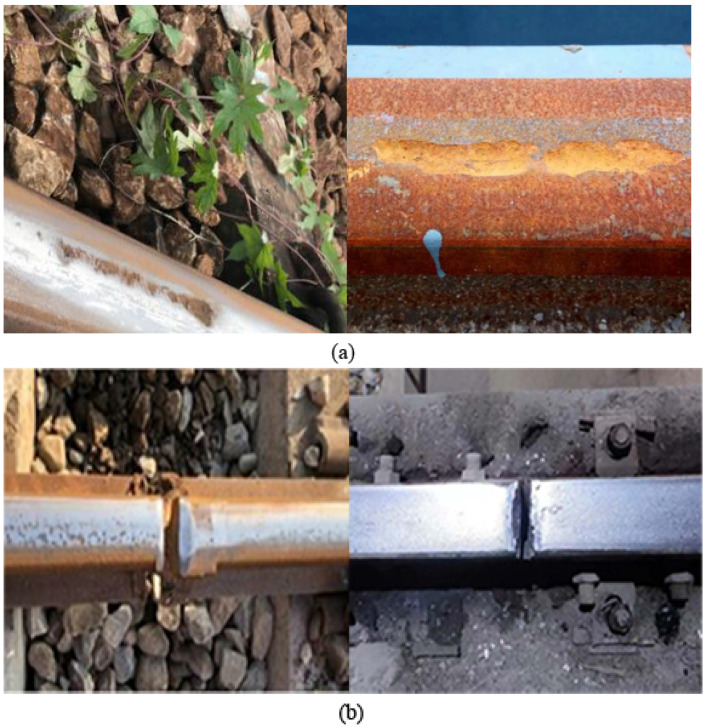
The resemblance between the characteristics of lifting equipment wheel–rail faults and railroad wheel–rail faults: (**a**) rail surface spalling. (**b**) rail cracks.

Many fault samples are necessary to train the YOLOv8 model to achieve optimal training performance. Nevertheless, the need for more fault samples from lifting equipment presents challenges in attaining satisfactory detection results through direct training from scratch. Transfer learning is a technique that utilizes the knowledge acquired from a known domain and applies it to the target domain. It enables the transfer of a trained network model from a large dataset to a new dataset, thus facilitating the reuse of network model parameters and weights in the new dataset. In order to overcome the problem of limited availability of wheel track image samples in lifting equipment, we propose utilizing the transfer learning method to improve the model’s performance. Figure 6 illustrates the resemblance between the characteristics of lifting equipment wheel–rail faults and railroad wheel–rail faults. Consequently, we employ a dataset of railroad wheel–rail faults obtained from the internet for pretraining. The collected dataset consists of 8400 images encompassing six types of faults: spalled rail treads, chewed rails, cracked rails, spalled wheel treads, broken fasteners and broken bolts. Similarly, we collected images of these six types of faults from the project site. After enhancement operations such as flipping, rotating and affine, the images from the project site were augmented to 300 images. Subsequently, we partition the images into training and test sets in an 8:2 ratio. It is important to note that all images are automatically resized to 640 × 640 pixels to improve the detection of small objects. Initially, we utilized the collected dataset to pre-train the enhanced model, leading to the acquisition of pretraining weights. Subsequently, we transferred the pre-training weights to our dataset for retraining to improve the model’s accuracy and generalization capabilities.

### 3.2. Experimental Platform Setup and Evaluation Indicators

The experiments detailed in this paper were conducted on the Ubuntu 20.04.3LTS operating system, utilizing an Intel^®^ Xeon^®^ Gold 5218R CPU and an NVIDIA GeForce RTX 3060 GPU with 12G of graphics memory. Python version 3.10 and the Pytorch 2.1.1 framework were used, along with acceleration libraries of CUDA 11.8 and CUDNN 8.9.7. The parameters of the backbone and the experimental training parameters can be found in Table 1 and Table 2.

The model metrics in this paper are evaluated using precision (P), recall (R), average precision (AP) and mean average precision (mAP). The following equations define the calculations for these metrics.

(12) $$P=\frac{\mathrm{TP}}{\left(\mathrm{TP}+\mathrm{FP}\right)}$$

(13) $$R=\frac{\mathrm{TP}}{\left(\mathrm{TP}+\mathrm{FN}\right)}$$

(14) $$\Omega_{\mathrm{AP}}=\int_{0}^{1}P\left(R\right)dR$$

(15) $$\Omega_{\mathrm{mAP}}=\frac{\sum_{i=1}^{k}\Omega_{{\mathrm{AP}}_{i}}}{k}$$

where TP refers to true positives, FP refers to false positives and FN refers to false negatives.

Model size, number of parameters, floating-point operations (FLOPs) and frames (FPS) were similarly used as measures of the model.

### 3.3. Ablation Experiment

An ablation study was conducted to demonstrate the improved performance of our proposed method for lifting equipment fault detection and identification, and the results are presented in Table 3. The results are compared with the ablation results obtained by incrementally adding classical FPN components such as BiFPN [38], ASFF [35] and DrFPN [39] to the YOLOv8 model.

The results show that standard YOLOv8 achieves a detection mAP of 76.2%. Integrating ST improves the mAP to 78.0%, and integrating specific FPN methods also leads to some improvement in the detection mAP. Our method outperforms YOLOv8 by achieving a 9.2% higher mAP on the dataset, demonstrating its impressive target detection and identification performance. Despite the significant increase in FLOPs caused by the inclusion of ST, the number of parameters and model size are reduced to some extent. While the proposed method experiences a significant increase in FLOPs due to the addition of ST, the modification of FPN leads to a substantial decrease in the model size and number of parameters, enabling easy deployment of the improved network on devices. Moreover, despite having an FPS of 26, which is only six units lower than the best-performing method, our proposed method still satisfies the real-time detection requirement.

### 3.4. Test Results

To showcase the advantages of the proposed method in detecting faults in lifting equipment, we conducted an evaluation using a dedicated dataset. This dataset contains six types of faults: rail surface spalling, rail gnawing, rail cracks, wheel surface spalling, fasteners broken and bolts broken. We collected images of these six types of faults from the project site. After enhancement operations such as flipping, rotating and affine, the images from the project site were augmented to 300 images. We compared its performance with the original YOLOv8, EfficientNet [40] and Fast R-CNN [41], among other models. Detecting faults in lifting equipment is a task involving multiple categories and objectives. The false detection rate and leakage rate stand as crucial indicators for evaluating the performance of the detection network. To validate the leakage detection capability of the proposed method in real-time lifting equipment fault detection, the logarithmic mean leakage detection rate is selected as the evaluation metric. The logarithmic mean leakage rate captures the relationship between each image’s FP and leakage rate. A lower FP rate corresponds to improved detection performance for lifting equipment faults. Table 4 presents a comparison of the evaluation metrics for each model in detecting faults in lifting equipment. Compared to various target detection algorithms, the enhanced detection algorithms exhibit superior precision compared to the models above. mAP surpasses the algorithms of EfficientNet, Fast R-CNN, YOLOv5, YOLOv6 and YOLOv8 by 3.3%, 4.5%, 3.2%, 1.6%, and 2.2%, respectively, while recall sees improvements of 2.0%, 3.7%, 3.3% and 3.3%. 3.7%, 3.3%, 2.9%, and 3.0%, respectively. In analyzing the detection accuracy across various fault types, the proposed method notably enhances the recognition accuracy of small targets, such as track tread and wheel tread spalling. At the same time, its performance is not notably outstanding in recognizing larger targets. This result demonstrates that the enhanced algorithm effectively enhances the detection accuracy of small targets and addresses challenges related to similar classifications.

We conducted a comparison and evaluation of the number of parameters and inference speed for several algorithms on images related to lifting equipment faults, as depicted in Table 5. Firstly, it is evident that the proposed method possesses a model size of 9.5 MB, making it easily deployable on a mobile platform, facilitating real-time capturing and recognition at the equipment system’s end. The number of parameters in the proposed method is lower than that of the other models. FLOPs of the proposed method amount to 22.8 G, representing only a 13.9 G increase compared to the best-performing YOLOv8. Based on these metrics, it becomes evident that our method exhibits faster training times, demands fewer hardware resources, and is readily applicable for generalization. However, an excessive reduction in parameters and computation may result in a reduction in the detection capability of the final trained model.

In summary, our method demonstrates high accuracy in multiscale target detection, effectively striking a balance between recognition accuracy and speed. The model is optimized for deployment on mobile terminals, showcasing practical applications.

**Figure 7:**
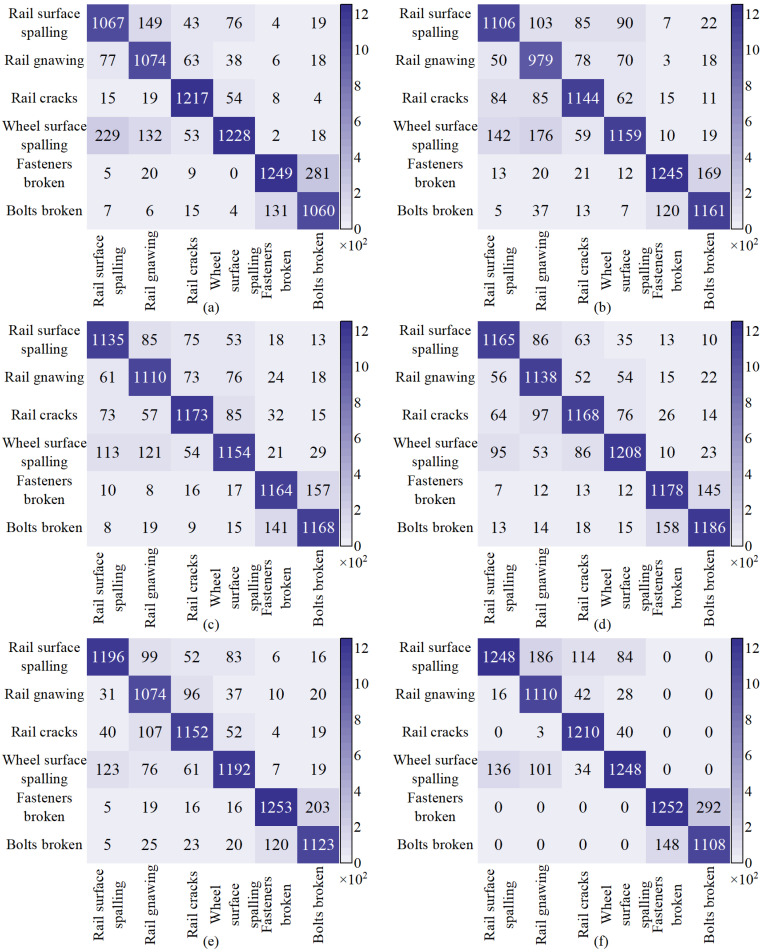
Confusion matrix of mainstream algorithms: (**a**) EfficentNet. (**b**) Faster R-CNN. (**c**) YOLOv5. (**d**) YOLOv6. (**e**) YOLOv8. (**f**) Ours.

The categorization of each class was visualized using the mainstream confusion matrix method, as illustrated in Figure 7. The data on the diagonal line signify the proportion of correctly categorized categories. This result reveals that the elevated FN category for lifting equipment faults implies the omission of a significant number of objects. The associated AP is likewise low. As evident in Figure 7, the outcomes of the proposed method exhibit mutual misclassification among rail surface spalling, rail gnawing, rail cracks and wheel surface spalling. However, they are not classified as fasteners broken and bolts broken. Rail and wheel surface spalling displayed the most pronounced mutual misclassification among these. Similarly, fasteners broken and bolts broken were also misclassified with each other, yet they were not categorized as other fault types. These results suggest that the proposed method excels in achieving comprehensive classification for faults with significant differences, while it may lack effectiveness in classifying faults of the same type. In contrast, other methods exhibit mutual misclassification for each fault type.

**Figure 8:**
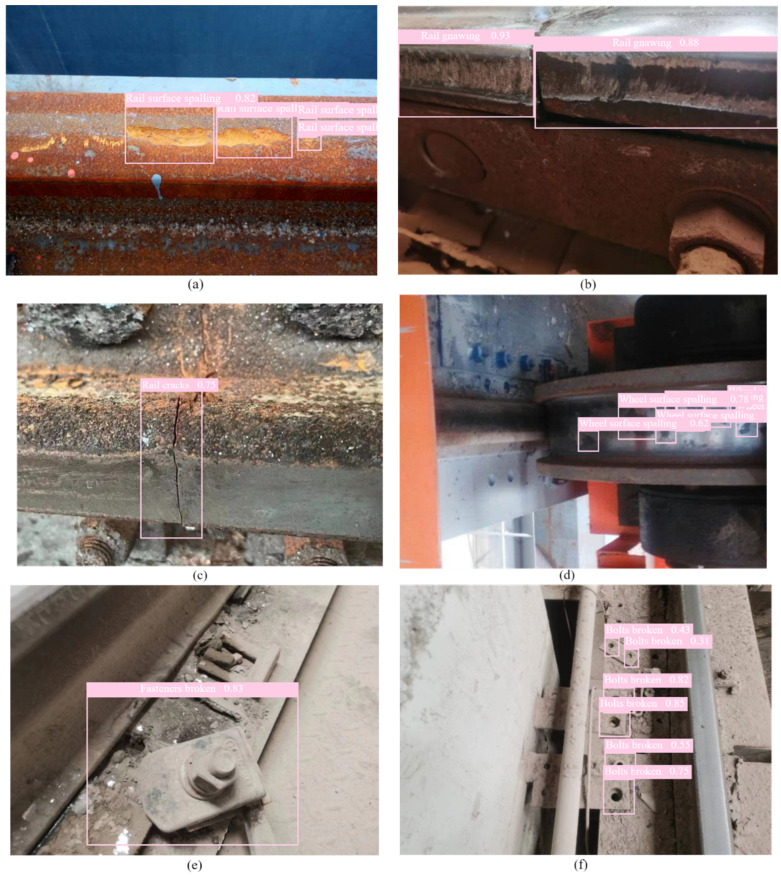
Detection results of the proposed method: (**a**) Rail surface spalling. (**b**) Rail gnawing. (**c**) Rail cracks. (**d**) Wheel surface spalling. (**e**) Fasteners broken. (**f**) Bolts broken.

Representative detection results of the proposed method are presented in Figure 8. Evidently, the proposed method successfully identifies small-sized faults in the scene with a high recognition accuracy. Therefore, the proposed method applies to various small and medium-sized objects, demonstrating the value of contextual knowledge in offering additional assistance. Furthermore, the proposed method exhibits outstanding performance in object categories characterized by significant scale variations, such as spalling and cracks. Thus, the proposed method can extract detailed low-level features for localization and high-level semantics for recognition.

## 4. Conclusions

Condition monitoring serves as a crucial foundation for ensuring the safe operation of special equipment. Machine vision fault detection stands out as the primary method for accurately determining the status of special equipment. Addressing the challenges posed by variable morphology and scale in special equipment faults, this paper adopts YOLOv8 as the algorithm’s baseline. It integrates the Swin Transformer backbone network to extract global features from the image and introduces the progressive feature pyramid network to enhance sensitivity to small target objects. Following enhancements, the mean average precision (mAP) of the algorithm rises from 83.2% to 85.4%, thereby improving the capability to detect faults in special equipment images. However, additional environmental factors were not thoroughly considered during the experiment. Consequently, in future research, the network structure and parameters of the algorithm will be further enhanced to account for more severe weather conditions, including heavy rainfall, heavy snow, high winds and low temperatures. This endeavor will contribute to enhancing the image recognition accuracy of special equipment, consequently improving the state awareness of special equipment.

## Figures and Tables

**Table 1:** The parameters of the backbone.

Layers	Repeats	Args	Output	Activation
Input	1		640 × 640 × 3	
Conv	1	[64, 3, 2]	320 × 320 × 16	SILU
Conv	1	[128, 3, 2]	160 × 160 × 32	SILU
Swin Transformer	3	[128]	160 × 160 × 32	
Conv	1	[256, 3, 2]	80 × 80 × 64	SILU
Swin Transformer	6	[256]	80 × 80 × 64	
Conv	1	[512, 3, 2]	40 × 40 × 128	SILU
Swin Transformer	6	[512]	40 × 40 × 128	
Conv	1	[1024, 3, 2]	20 × 20 × 256	SILU
Swin Transformer	3	[1024]	20 × 20 × 256	
SPPF	1	[1024, 5, 1]	20 × 20 × 256	

**Table 2:** Experimental training parameters.

Parameters	Values
Learning rate	0.01
Optimizer	Adam
Batch	8
Epochs	300

**Table 3:** Ablation experiment results.

Method	Model (MB)	Parameters (M)	FLOPs (G)	FPS	P (%)	R (%)	mAP (%)
YOLOv8	12.3	3.2	8.9	34	75.0	76.3	76.2
YOLOv8 + ST	12.0	3.0	24.3	33	77.7	77.0	78.0
YOLOv8 + BiFPN	12.3	3.2	8.9	33	78.0	76.8	79.0
YOLOv8 + ST + BiFPN	12.1	3.0	24.4	32	79.0	79.1	79.3
YOLOv8 + ASFF	17.5	4.5	11.0	22	82.0	82.0	81.6
YOLOv8 + ST + ASFF	17.3	4.4	26.5	20	84.1	83.2	82.3
YOLOv8 + DrFPN	14.9	3.9	7.5	33	85.8	83.0	84.0
YOLOv8 + ST + DrFPN	14.4	3.7	23.0	27	87.3	85.1	85.3
Ours	9.5	2.3	22.8	26	87.8	85.3	85.4

**Table 4:** Mainstream algorithm comparison results.

Method	AP (%)						mAP (%)	P (%)	R (%)
	Rail Surface Spalling	Rail Gnawing	Rail Cracks	Wheel Surface Spalling	Fasteners Broken	Bolts Broken			
EfficentNet	76.2	76.7	86.9	87.7	89.2	75.7	82.1	82.5	83.3
Faster R-CNN	79.0	69.9	81.7	82.8	88.9	82.9	80.9	80.5	81.6
YOLOv5	81.1	79.3	83.8	82.4	83.1	83.4	82.2	81.5	82.0
YOLOv6	83.2	81.3	83.4	86.3	84.1	84.7	83.8	83.7	82.4
YOLOv8	85.4	76.7	82.3	85.1	89.5	80.2	83.2	82.0	82.3
Ours	89.1	79.3	86.4	89.1	89.4	79.1	85.4	87.8	85.3

**Table 5:** Number of parameters and inference speed of mainstream algorithms.

Method	Model (MB)	Params (M)	FLOPs (G)	FPS
EfficentNet	15.2	3.8	25.1	19
Faster R-CNN	119.5	28.5	939.6	12
YOLOv5	11.2	4.6	10.8	24
YOLOv6	10.3	4.3	11.06	29
YOLOv8	12.3	3.2	8.9	34
Ours	9.5	2.3	22.8	26

## Data Availability

The raw/processed data required to reproduce these findings cannot be shared at this time as the data also form part of an ongoing study.
